# A Single-Run Next-Generation Sequencing (NGS) Assay for the Simultaneous Detection of Both Gene Mutations and Large Chromosomal Abnormalities in Patients with Myelodysplastic Syndromes (MDS) and Related Myeloid Neoplasms

**DOI:** 10.3390/cancers13081947

**Published:** 2021-04-18

**Authors:** Alessandro Liquori, Iván Lesende, Laura Palomo, Gayane Avetisyan, Mariam Ibáñez, Elisa González-Romero, Mireia Boluda-Navarro, Mireya Morote-Faubel, Cristian Garcia-Ruiz, Cristina Martinez-Valiente, Marta Santiago-Balsera, Inés Gomez-Seguí, Alejandra Sanjuan-Pla, Miguel A. Sanz, Guillermo Sanz, Francesc Solé, Esperanza Such, José Cervera

**Affiliations:** 1 Hematology Research Group, Instituto de Investigación Sanitaria La Fe, 46026 Valencia, Spain; gayane_avetisyan@iislafe.es (G.A.); ibanyez_marcom@gva.es (M.I.); elisa_gonzalez@iislafe.es (E.G.-R.); mireia_boluda@iislafe.es (M.B.-N.); mireya_morote@iislafe.es (M.M.-F.); cristian_garcia_ruiz@iislafe.es (C.G.-R.); cristina_martinez@iislafe.es (C.M.-V.); santiago_marbal@gva.es (M.S.-B.); alejandra_sanjuan@iislafe.es (A.S.-P.); Miguel.Sanz@uv.es (M.A.S.); such_esp@gva.es (E.S.); 2 Centro de Investigación Biomédica en Red de Cáncer (CIBERONC), 28029 Madrid, Spain; gomez_ine@gva.es (I.G.-S.); sanz_gui@gva.es (G.S.); 3 Universidade de A Coruña (UDC), 15006 A Coruña, Spain; Biologousc@hotmail.com; 4 MDS Research Group, Institut de Recerca Contra la Leucèmia Josep Carreras, Institut Català d’Oncologia-Hospital Germans Trias i Pujol, Universitat Autònoma de Barcelona, 08916 Badalona, Spain; lpalomo@carrerasresearch.org (L.P.); fsole@carrerasresearch.org (F.S.); 5 Department of Hematology, Hospital Universitario y Politécnico La Fe, 46026 Valencia, Spain; 6 Genetics Unit, Hospital Universitario y Politécnico La Fe, 46026 Valencia, Spain

**Keywords:** myelodysplastic syndromes, cytogenetics, next-generation sequencing, myeloid neoplasm, SNP array, karyotype

## Abstract

**Simple Summary:**

Chromosomal abnormalities and somatic mutations are found in patients with myelodysplastic syndromes (MDS) and myelodysplastic/myeloproliferative neoplasms (MDS/MPN) in around 50–80% of cases. The identification of these alterations is important for the accurate diagnosis and prognostic classification of these patients. Often, an apparently normal or failed karyotype might lead to an inadequate estimation of the prognostic risk, and several strategies should be combined to solve these cases. The aim of this study was to introduce a novel next-generation sequencing (NGS)-based strategy for the simultaneous detection of all the clinically relevant genetic alterations associated with these disorders. We validated this approach on a large cohort of patients by comparing our findings with those obtained with standard-of-care methods (i.e., karyotype and SNP-arrays). We show that our platform represents a significant improvement on current strategies in defining diagnosis and risk stratification of patients with MDS and myeloid-related disorders.

**Abstract:**

Myelodysplastic syndromes (MDS) and myelodysplastic/myeloproliferative neoplasms are clonal disorders that share most of their cytogenetic and molecular alterations. Despite the increased knowledge of the prognostic importance of genetics in these malignancies, next-generation sequencing (NGS) has not been incorporated into clinical practice in a validated manner, and the conventional karyotype remains mandatory in the evaluation of suspected cases. However, non-informative cytogenetics might lead to an inadequate estimation of the prognostic risk. Here, we present a novel targeted NGS-based assay for the simultaneous detection of all the clinically relevant genetic alterations associated with these disorders. We validated this platform in a large cohort of patients by performing a one-to-one comparison with the lesions from karyotype and single-nucleotide polymorphism (SNP) arrays. Our strategy demonstrated an approximately 97% concordance with standard clinical assays, showing sensitivity at least equivalent to that of SNP arrays and higher than that of conventional cytogenetics. In addition, this NGS assay was able to identify both copy-neutral loss of heterozygosity events distributed genome-wide and copy number alterations, as well as somatic mutations within significant driver genes. In summary, we show a novel NGS platform that represents a significant improvement to current strategies in defining diagnosis and risk stratification of patients with MDS and myeloid-related disorders.

## 1. Introduction

Myelodysplastic syndromes (MDS) and myelodysplastic/myeloproliferative neoplasms (MDS/MPN), including chronic myelomonocytic leukemia (CMML), are clonal myeloid disorders whose classification has been updated in the last World Health Organization (WHO) revision [1,2]. Despite presenting distinct hematologic and morphological features, this heterogeneous group of hematopoietic stem cell malignancies shares an increased risk of progression to acute myeloid leukemia (AML), as well as most of the cytogenetic and molecular alterations.

Cytogenetic abnormalities are found in ~50% and 20–30% of MDS and CMML patients, respectively [3,4,5]. These are mainly unbalanced changes, leading to copy number alterations (CNAs) as a result of a partial or complete loss or gain of chromosome material [3,5]. Common alterations include abnormalities of chromosome 7, trisomy 8 and 21, −Y, and complex karyotypes [3,4]. Other CNAs are more typical of MDS, such as del(5q), del(20q), del(12p), and del(17p)/i(17)(q10), while reciprocal translocations are rare (~2–3%) [3].

Somatic mutations are identified in 78–90% and up to 95% of MDS and CMML patients, respectively [6,7,8,9]. Over 40 genes are recurrently mutated in both disorders, but with different frequencies and patterns of co-occurrence and mutual exclusivity of mutations. Altered genes belong to several functional pathways, and RNA splicing factors (*SRSF2, SF3B1, ZRSR2,* and *U2AF1*) and epigenetic regulators (*TET2, DNMT3A, IDH1/2, ASXL1,* and *EZH2*) are the most frequent [10].

Several prognostic scoring systems have been hitherto developed to predict MDS and CMML outcomes and improve the diagnosis, prognosis, and treatment strategies of the patients [11,12,13,14,15,16]. Cytogenetics has been integrated as one of the most influential prognostic parameters in standard MDS and CMML stratification models, such as the Revised International Prognostic Scoring System (IPSS-R) and the CMML-specific prognostic scoring system (CPSS), respectively [13,16]. Recently, updated molecular versions of each model have been proposed by integrating mutation data from *EZH2*, *SF3B1,* and *TP53* to adjust IPSS-R (IPSS-Rm) [17] as well as *RUNX1, NRAS, SETBP1,* and *ASXL1* to the CPSS model (CPSS-Mol) [8].

Despite the increased knowledge of the prognostic relevance of genetics in these malignancies, the molecular versions of these score systems have not completely replaced the use of the original models, mainly because NGS has not been incorporated into clinical practice in a validated manner. In contrast, conventional karyotype analysis still remains mandatory in the evaluation of suspected MDS and related neoplasms [1,18,19]. However, non-informative cytogenetics due to an apparently normal or failed karyotype might lead to an inadequate estimation of the prognostic risk. Single-nucleotide polymorphism (SNP) array-based platforms can be used in these cases to increase the diagnostic yield by detecting not only CNAs, which are as small as 20 kb in size, but also copy-neutral loss of heterozygosity (cnLOH) [20,21,22]. Nevertheless, with the exception of focal lesions involving relevant driver genes (e.g., *TET2, EZH2*, *TP53*) [10], no prognostic significance has been established for the majority of reported cryptic/submicroscopic CNAs, thus challenging the clinical interpretation of these findings [23,24].

In this study, we aimed to develop a single-run targeted NGS-based assay that allows the detection of most of the genome-wide MDS and CMML-related CNAs and cnLOH, as well as of the recurrent somatic mutations reported to have clinical significance in these neoplasms. To validate this strategy, we studied 60 patients in parallel by using our NGS platform, conventional cytogenetics, and SNP arrays.

## 2. Materials and Methods

### 2.1. Patients and Samples

A total of 60 patients diagnosed with primary MDS or closely related neoplasms (including CMML and other MDS/MPN) at the University Hospital La Fe (HULAFE) in Valencia (Spain) between 2014 and 2017 constituted the basis of this retrospective non-interventional study. Diagnoses were made according to the 2016 WHO revision [1,2]. Prognosis of MDS and CMML cases was estimated according to the IPSS-R [13] and CPSS [16] scores, respectively, and adjusted with molecular data (IPSS-Rm [25] for MDS patients and CPSS-Mol [8] for CMML) (Table 1). Samples were obtained with written informed consent in accordance with the Declaration of Helsinki and the approval of the review board of Bioethics and Medical Research at HULAFE.

DNA from patients was isolated from bone marrow mononuclear cells obtained at the time of diagnosis using a QIAamp^®^ DNA Mini kit (QIAGEN). Germline samples were not available. DNA from B-lymphocytes of HapMap NA12144 (Coriell Institute) was used as a control. All of the analyses performed in this study (conventional cytogenetics, NGS, and molecular karyotyping) were carried out on the same patient DNA specimens.

### 2.2. Conventional Cytogenetics

Cytogenetic studies were performed according to standard procedures. The chromosomes were stained by G-banding and the karyotypes were reported according to the International System for Human Cytogenetic Nomenclature (ISCN, 2016 recommendations) [26]. Fluorescence in situ hybridization (FISH) was performed to confirm selected CNAs (e.g., del(5q), del(7q) or +8) by using chromosome-specific probes (Abbott. Downers Grove, IL). The interphase FISH cut-off percentages were 5% for common alterations, including del(5q), del(7q), and +8, and 7% for gene deletion (e.g., *MECOM*) or amplification (e.g., *CKS1B*) [27].

### 2.3. Molecular Karyotyping

SNP arrays were performed with an Affymetrix CytoScan^®^ HD Array according to the manufacturer’s protocol (Affymetrix, Thermo Fisher Scientific, Santa Clara, CA, USA). CNAs and cnLOH analysis were performed using the Chromosome Analysis Suite (ChAS) software (Affymetrix) v3.1 (Thermo Fisher Scientific, Santa Clara, CA, USA). The filters applied for the detection of CNAs consisted of ≥10 consecutive markers in a region of at least 10 kb, and ≥200 markers in at least 10 Mb for regions of cnLOH. The human reference GRCh37/hg19 assembly was used for alignment. All CNAs found were compared with the Database of Genomic Variants (http://dgv.tcag.ca/dgv/app/home, accessed on 25 August 2020) in order to distinguish the constitutional copy number alterations present in healthy controls from somatic alterations. The clinical significance of CNAs and cnLOH was established according to previous literature [23,24].

### 2.4. Library Preparation and Sequencing

A custom target enrichment library design was performed using the SureDesign (Agilent Technologies, Santa Clara, CA, USA) web application. RNA probes were manufactured to capture a panel of 40 genes selected according to the Spanish Group of MDS (GESMD) guidelines [18] (total probe size: 213,846 kb; Table S1). This custom design was combined with the “*OneSeq 1 Mb CNV Backbone*” commercial design (Agilent Technologies) to enable the detection of CNAs and cnLOH with resolutions of 1 and 10 Mb, respectively (total probe size: 2.674 Mb). Sequencing libraries were prepared using the “SureSelect^QXT^ Library Prep” protocol (Agilent Technologies) starting from 50 ng of DNA and a unique 90-minute capture step. The quality and concentration of DNA samples and libraries were assessed with the 4200 TapeStation System (Agilent Technologies), and 10 sample pools were sequenced (75 × 2 cycles) on a NextSeq 500 (Illumina, San Diego, CA, USA). The mean number of reads in .bam files passing the mapping quality filters per sample was 14,226,190 ± 2,825,217 and had a 319 ± 133x average depth (Table S2). Ten (17%) samples were randomly selected to perform a 50 and 75% subsampling of available reads (https://github.com/lh3/seqtk, accessed on 10 April 2021) for further validation of the strategy. 

### 2.5. Bioinformatic Data Analysis

#### 2.5.1. Somatic Mutations

Data processing was performed using the Agilent SureCall 4.0 software with the default configuration. Sequence reads were aligned against the human reference GRCh37/hg19 genome by using the BWA-MEM algorithm [28] for Illumina data. The BAQ/SNP caller [29] (SAMtools) was used to identify single-nucleotide variants (SNVs) and indels. Each putative variant was annotated with wANNOVAR (http://wannovar.wglab.org/, accessed on 30 April 2020) using the Short Genetic Variations database dbSNP147, gnomAD, 1000 Genomes Project [30], ClinVar [31], and COSMIC (https://cancer.sanger.ac.uk/cosmic, accessed on 30 April 2020). In the absence of matched control samples, the criteria to distinguish somatic from germline variants with confidence were mainly based on information from available population databases (minor allele frequency, MAF > 0.01), previous literature on the genomic landscape of myeloid neoplasms, and frequencies of variant reads [6,7]. The pathogenicity of variants was assessed according to the GESMD guidelines [18]. All putative pathogenic and likely pathogenic variants were visually revised in the Integrative Genomics Viewer (IGV). Among them, 52 (58%) variants were randomly selected for further validation by using the Myeloid Solution^TM^ (SOPHiA GENETICS) commercial design and the SOPHiA DDM^®^ analytical platform (Table S3).

#### 2.5.2. Copy Number Alterations

CNA (i.e., deletion and duplication lesions) analysis was performed on the “*CNV Calls tab*” obtained from the use of the DECoN (Detection of Exon Copy Number variants) algorithm [32]. This tool was installed in a Linux environment and run according to the developers’ instructions. Analysis was performed by separating patients into two groups depending on sex with a median correlation threshold of 0.97 (range: 0.74–0.98). Furthermore, CNAs were also called by using the Ginkgo open-source web platform (http://qb.cshl.edu/ginkgo, accessed on 20 August 2020) [33]. Briefly, sample .bed files converted from .bam by Galaxy (https://usegalaxy.org/, accessed on 20 August 2020) were uploaded on the web platform and analyzed using default criteria with a 1 Mb *General Binning Option*. Samples were organized in hierarchical trees and heat maps depending on their copy number profiles.

To improve the accuracy of CNA detection, including that of cnLOH, the B-allele frequency (BAF) was calculated on 172,470 genome-wide-distributed SNPs (Table S4). These were chosen to have high MAFs (range: 0.40–0.45) in multiple human populations in order to increase the probability of heterozygosity. To compute the BAF, the counts of sequencing reads showing the most commonly occurring alleles were divided by the counts of sequencing reads showing the two most common alleles at a specific SNP locus for a specific sample. CNAs and cnLOH events were manually assigned by comparing all CNA calls from DECoN and Ginkgo with the BAF plots in the corresponding regions.

HapMap NA12144 and subsampled specimens were analyzed with the same pipelines, and the results were compared with those of the previous literature (Table S5) and with findings obtained from conventional analyses, respectively. 

## 3. Results

### 3.1. Detection of Somatic Mutations

**Figure 1:**
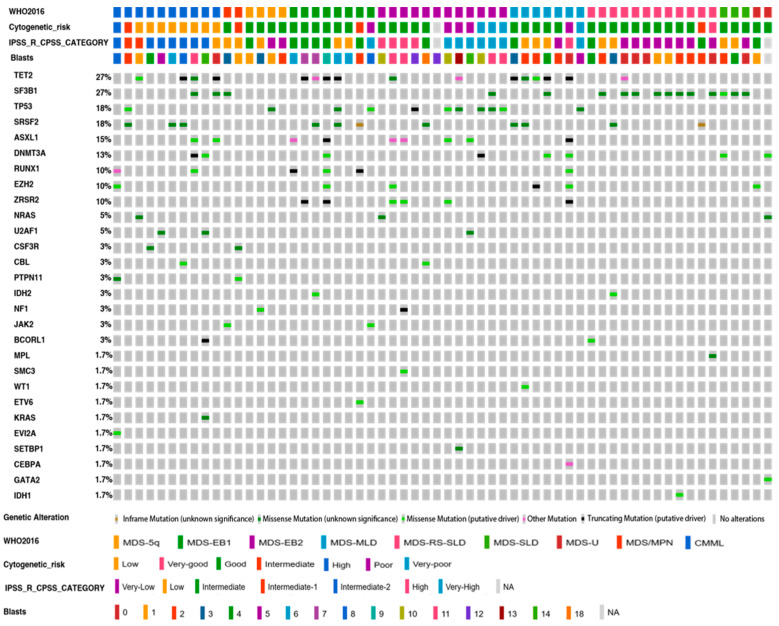
Distribution of clinically relevant variants of significantly mutated genes in 60 MDS/CMML cases. Pathogenic and likely pathogenic variants (SNVs and indels) identified in 28 out of the 40 genes analyzed are shown. Of the 60 samples evaluated, 54 (90%) had at least one mutation in one of the listed genes. The WHO 2016 classification, the cytogenetic risk, the IPSS-R or CPSS category, and the number of blasts for each patient are shown at the top of the chart. The color of the mutation depends on its functional type. The heatmap was made with the OncoPrinter tool (https://www.cbioportal.org/visualize, accessed on: 10 April 2021).

We sequenced 40 genes across 60 patients with MDS and closely related neoplasms, resulting in 2,221 variants, of which 696 were unique. After following the variant filtering workflow [18], we selected 127 SNVs/indels. Among them, 66 had been previously reported in myeloid malignancies and categorized as pathogenic, while 23 were classified as likely pathogenic (Table S3). We randomly selected 52 variants for further validation by using another targeted design, confirming all of them (Table S3). Deleterious variants were located in 28 genes (Figure 1), and *TET2* and *SF3B1* were the most frequently mutated in the cohort (27%), followed by *TP53* and *SRSF2* (18%). Another five genes (*ASXL1, DNMT3A, RUNX1, EZH2,* and *ZRSR2*) were mutated in more than 10% of patients, and the rest in less than 5%, with only one mutation detected in 18 of them.

**Figure 2:**
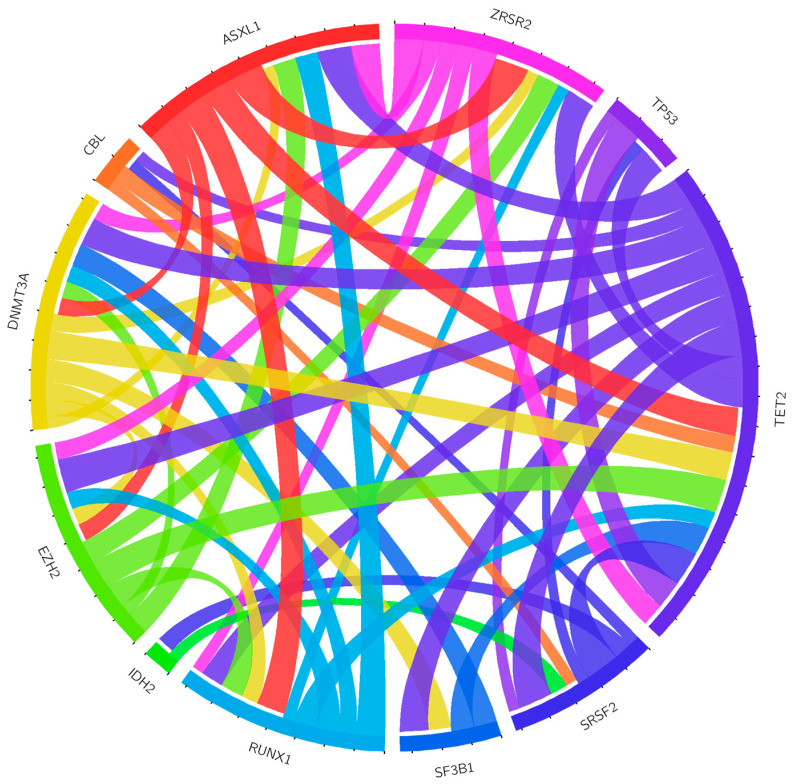
Patterns of co-occurrence among clinically relevant variants. Circos diagram (http://circos.ca/, accessed on: 25 February 2021) showing co-occurring mutational events within the whole cohort. Colored lines are used for different genes and refer to one mutation each.

The splicing factor genes, *SRSF2* and *SF3B1*, had the most common hotspots (P95H, L, R, P95_103del, P95delinsRP; *n* = 11 cases) and mutations (K700E; *n* = 9) in the cohort, respectively (Table S3). Furthermore, we searched for pairwise gene associations (*n* = 378) by detaching the co-mutation of *ASXL1* and *ZRSR2* (q = 0.049, Benjamini–Hochberg correction procedure), in addition to 16 other positive and negative correlations (*p* < 0.05, one-sided Fisher Exact test) (Figure 2 and Table S6).

When the variant distribution was analyzed, 54 (90%) out of 60 patients harbored at least one mutation, with a median of 2 (range 0–7) per sample. The mean number of mutations correlated with the WHO subtypes and IPSS-R/CPSS risk categories, which were higher in high-risk groups (Figure 1).

### 3.2. Detection of Copy Number Changes from Conventional Cytogenetics and SNP Arrays

Conventional cytogenetics was performed in all of the patients of the cohort, which showed a normal karyotype, chromosomal alterations, and an unsuccessful karyotype in 62% (*n* = 37), 37% (*n* = 22), and 1% (*n* = 1) of cases, respectively (Table 1). One patient harboring a germinal Robertsonian translocation (45,XX,rob(13;14)(q10;q10)c[20]) was included in the normal karyotype group.

In addition, we performed SNP arrays to extend the detection of CNAs to cryptic alterations (<5 Mb). These confirmed all but three alterations (86%, 19 out of 22) that were previously identified with conventional cytogenetics, including a subclonal trisomy 8 and 21, as well as an i(17)(q10), which was instead identified as a del(17p). Interestingly, the SNP arrays reclassified four cases with monosomy 7 as del(7q) and identified 12 additional lesions (from 4.9 to 60 Mb in size), including deletions of 5q (*n* = 3), 7q (*n* = 1), 12p (*n* = 4), 17p (*n* = 2), and 20q (*n* = 2) (Table 2, Tables S7 and S8). In addition, three high-risk MDS patients (5%) displayed chromothripsis involving chromosomes 6p, 12, 16p, 18q, 19p, and 21q and affecting the *ETV6, U2AF1,* and *RUNX1* genes, among others. In all of these cases, the *TP53* gene was mutated.

### 3.3. Detection of Copy Number Changes from NGS Data

We assessed the feasibility of our NGS platform in detecting CNAs by blindly examining the performance of an in-house workflow based on the use of two user-friendly bioinformatic tools, DECoN and Ginkgo, separately and in combination (Tables S7, S9 and S10). 

In the case of patients with aberrant cytogenetics (*n* = 22, 37%), NGS confirmed 89% (23 out of 26) of the CNAs identified by conventional cytogenetics, including alterations of chromosome 7 (*n* = 9), deletions of 5q (*n* = 8), 12p (*n* = 1), loss of Y (*n* = 1), trisomy 8 (*n* = 2), and trisomy 21 (*n* = 2). Only three exceptions were observed, including a 16 Mb del(20q) and two subclonal alterations involving trisomy 8 and an aberrant chromosome 1. Although validated by FISH, in 5% and 7.5% of the nuclei, respectively, neither subclonal alteration was observed with the SNP arrays, likely due to their sensitivity (>20% of cells) [22].

When normal-karyotype patients were evaluated, the NGS platform agreed with conventional cytogenetics for 28 (76%) of them, detaching cryptic alterations in nine (24%) cases and bringing the number of patients with CNAs from 22 to 31.

From the comparison of the NGS results with those obtained by using SNP arrays, 51 CNAs were identified with both assays; 17 were uniquely identified by the SNP arrays and three uniquely by our NGS platform (Table 2 and Table S7). As a whole, 89% (*n* = 31/35) and 67% (*n* = 22/33) of concordance were observed for clinically relevant lesions and CNAs with unknown prognostic significance, respectively. However, discrepancies were seen in six patients, which concerned two del(12p) and del(20q), one del(17p), and a trisomy 21. Among them, those involving chromosomes 12 and 21 observed in the two MDS-EB2 patients deserve to be highlighted (Figure S1). In the first case, a telomeric duplication (12p13.33-p13.31, 7.5 Mb) and a flanking deletion (12p13.31-p12.3, 7.9 Mb), including the *ETV6* gene, were identified exclusively with the NGS and SNP arrays, respectively. In the second case, NGS identified a trisomy 21, whereas the SNP arrays detected a dup(21q21.2-q22.3) interspaced by a 1-Mb 21q21.3 deletion. Other discrepancies (*n* = 12) observed across the six patients corresponded exclusively to CNAs with unknown prognostic significance, ranging from 3.2 to 21.8 Mb and mainly involving chromosomes 19 (*n* = 3) and 5p (*n* = 2).

It is of note that the NGS assays confirmed all of the chromotripsis events observed with the SNP arrays (Figure S2), with only the exception of that involving chromosome 21q. Finally, both DECoN and Ginkgo agreed in identifying a normal karyotype in the case with unsuccessful cytogenetics.

### 3.4. Detection of Copy-Neutral Loss of Heterozygosity Lesions from SNP Arrays and NGS Data

Copy-neutral LOH events were identified in 12 (20%) patients with the SNP arrays, including eight with normal cytogenetics (Figure S3). Uniparental disomy of chromosomes 7q (*n* = 4) and 4q (*n* = 2) was the most frequent alteration.

We also explored the possibility of identifying cnLOH events with our NGS assay. For each chromosome, we plotted the BAF of the targeted germline SNPs and excluded the presence of copy number changes by using the outputs of DECoN and Ginkgo. Fourteen cnLOH lesions with a median size of 59.2 Mb (range: 10.2–116 Mb) were detected in the same 12 patients with a 100% concordance with the SNP array results (Figure S3 and Table S11).

### 3.5. Correlation between CNAs and Mutations Detected by NGS

We analyzed the correlation among the different types of genomic lesions detected with NGS. In total, 57 (95%) patients harbored at least one cytogenetic (*n* = 3), molecular (*n* = 26), or both types of alterations (*n* = 28). Only three low-risk MDS patients did not show any genomic lesions.

Focusing on the patients harboring CNAs or cnLOH and mutations within the same locus (Table S12), we found that all of the patients with a deletion (*n* = 3) or cnLOH (*n* = 1) of chromosome 17p had a *TP53* mutation, likely within the same clone (ρ = 0.86, Pearson correlation). This was also observed in three (38%) out of eight cases with aberrant chromosome 7, where *EZH2* mutations co-occurred with monosomy (two out of four cases) and 7q cnLOH (one out of four cases) (ρ = 0.99, Pearson correlation). In addition, the cnLOH of 2p (*n* = 1) and 4q (*n* = 2) correlated with *DNMT3A* and *TET2* (one out of two cases) mutations, respectively.

### 3.6. Clonality Studies and Breakpoint Definition of Del(5q), Del(7q), and Monosomy 7

**Figure 3:**
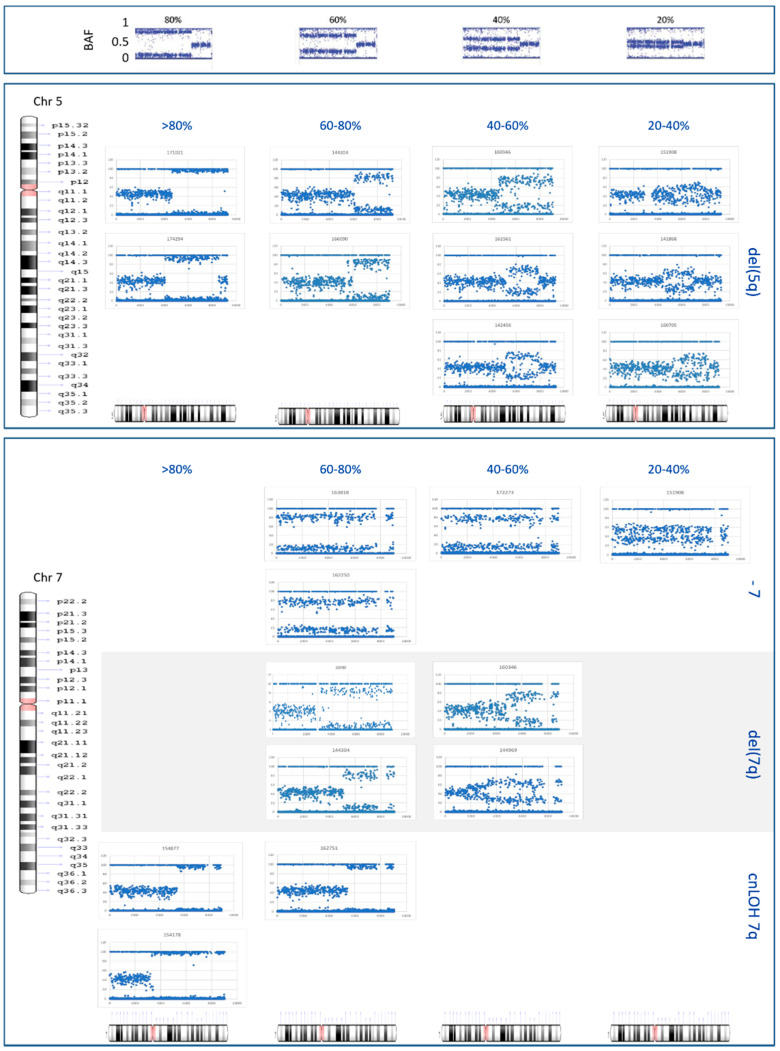
B allele frequency (BAF) plots for regions of CNAs and cnLOH within chromosomes 5 and 7. The BAF plots reported by Song Sarah et al [34] as a result of a dilution series of tumor DNA in normal samples are shown at the top of this chart. These were used to establish the tumor burden of alterations found in our cohort. CNAs and cnLOH identified within chromosome 5 (i.e., del(5q); in the medium chart) and 7 (i.e., monosomy 7, del(7q), and cnLOH of 7q; in the bottom chart) were organized in four subgroups depending on the level of separation of the BAF. This diagram was also used to establish the breakpoints of alterations for each patient. Deletions smaller than 10 Mb (i.e., del(5q) in #144969 and del(7q) in #144206) are not shown.

To establish whether a patient belongs to the MDS with isolated del(5q) category, according to the WHO classification, the presence of the del(5q) and the absence of chromosome 7 alterations should be assessed [1,2]. Therefore, we investigated samples carrying lesions in these chromosomes as a pretense for exploring the detection limit of our NGS strategy. In all of the patients where del(5q) (*n* = 11) and alterations of chromosome 7 (*n* = 12) were detected, these were found in >20% of cells. In addition, differences in the tumor burden were observed among samples (Figure 3 and Table S13).

In the case of del(5q), lesions ranged from 5.6 to 87 Mb, where 60 Mb was the median size. The breakpoints identified with NGS mainly coincided with those detected with the SNP arrays, with a ~200 kb median shift in the genomic position (range: 0–5 Mb). It is of note that two 60 Mb del(5)(q23.1q35.3) and one 5.6 Mb del(5)(q33.2q34) were identified with NGS as well as with the SNP arrays, but not with conventional cytogenetics. Unlike the first two lesions, the smallest deletion was not confirmed by FISH, probably due to the location of the probe used (i.e., 5q31.2). 

In addition, NGS redefined four monosomy 7 instances as one 7q cnLOH and three del(7q), and also identified two novel 7q cnLOH and one del(7q) events in two normal and one double-altered karyotype, respectively. These findings were further confirmed by SNP arrays with breakpoints shifting within a region of up to 42 Mb (median, 100 kb) and by FISH. Lesions of 7q ranged from 9.9 to 96 Mb (median, 64 Mb), where del(7q) events were the smallest and the largest alteration in the region. These started at 7q22 (*n* = 3), 7q21 (*n* = 2), or 7q11 (*n* = 2) and extended up to the telomere.

### 3.7. Patient Classification after NGS Finding Integration

**Figure 4:**
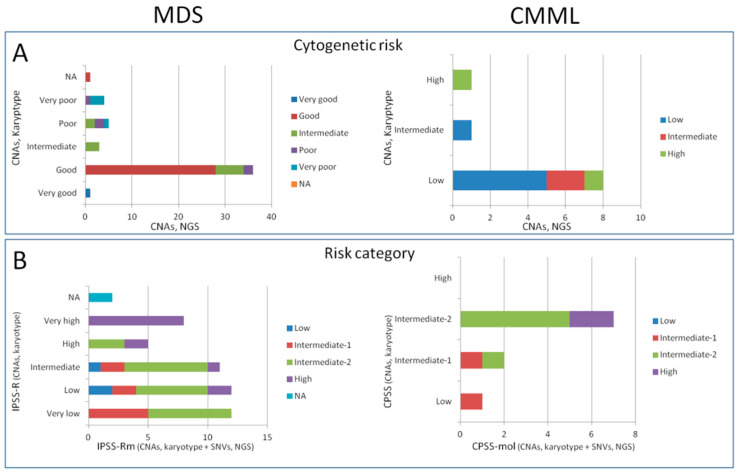
Distribution of changes in cytogenetic risk (**A**) and risk categories (**B**) across the original and molecular versions of IPSS-R and CPSS models when the results of NGS were used instead of conventional cytogenetics. This diagram shows the changes observed (upstage or downstage) across all considered models. The Y axis represents the original cytogenetic risk and risk category in the charts at the top (**A**) and at the bottom (**B**), respectively. The X axis represents the percentage of patients in each risk category found through the use of NGS. For example, among MDS patients with good cytogenetic risk according to conventional cytogenetics, ~78% still remain in this category, whereas 17% and 5% shift to intermediate and poor risk, respectively, when results from NGS are used. Abbreviations: NA, not available; CNAs, copy number alterations; SNVs, single-nucleotide variants; NGS, next-generation sequencing. IPSS-R, Revised International Prognostic Scoring System; IPSS-R, molecular IPSS-R; CPSS, CMML-specific prognostic scoring system, CPSS-mol, molecular CPSS.

After testing the performance of our NGS strategy, we assessed the clinical utility of this platform as a diagnostic and prognostic tool. Clinically significant CNAs detected with NGS were used to classify MDS and CMML patients according to the IPSS-R [13] and CPSS [16] scoring systems, respectively. Cytogenetic risk categories were confirmed in 74% (*n* = 37/50) of MDS and 60% (*n* = 6/10) of CMML cases. In the rest of the cohort, patients were upstaged or downstaged into other risk categories (Figure 4A and Table S14).

When molecular data were used with conventional cytogenetic data to establish patient risk stratification according to the novel prognostic classifications [8,17], the percentage of cases in which the result of the standard classification was confirmed decreased to 42% (*n* = 21/50) of MDS, although this remained unchanged for CMML (60%, *n* = 6) (Figure 4B). All MDS patients within the very high subgroup remained in the same category, whereas most of the changes were observed within the low-risk subgroups. Except for two cases, the rest of the patients were upstaged within the intermediate-2 (*n* = 13), intermediate-1 (*n* = 7), and high-risk categories (*n* = 2).

By contrast, no significant changes with the molecular version of the prognostic scores were reported when exclusively NGS data were used. Four MDS cases (8%) made exceptions, since a one-category change was observed in both directions between the intermediate-1 and -2 subgroups, as well as between the intermediate-2 and high-risk subgroups. In the case of CMML, an intermediate-1 and an intermediate-2 patient were downstaged and upstaged to the low- and high-risk groups, respectively. It is of note that the observed downgrades were due to the reclassification of a monosomy 7 as one 7q cnLOH and the oversight of a subclonal del(1)(p10) in an MDS and CMML patient, respectively.

## 4. Discussion

In this study, we described a novel single-run targeted NGS-based assay for the diagnosis and prognostic risk stratification of patients with MDS and related neoplasms. This strategy covers all of the clinically relevant genetic alterations, with the exception of translocations, which were excluded due to their rarity and uncertain prognostic significance in these malignancies [35,36]. We demonstrated the ability of our platform to identify CNAs and cnLOH events in a single experiment with a sensitivity at least equivalent to that of SNP arrays, in addition to somatic mutations within significant driver genes.

Recently, other NGS assays have been developed for the comprehensive genetic diagnosis of hematological neoplasms, such as MDS [37,38], AML [37,39,40], and multiple myeloma [41]. In our method, a unique capture protocol combining a commercial and a custom backbone was performed by using commercially available reagents and kits. Libraries were manually prepared and a benchtop device was chosen for sequencing. Raw data were obtained in a one-week timeframe. Strikingly, no specific bioinformatic algorithms were developed for the analysis of sequencing data; rather, we based our approach on the employment of free user-friendly tools that do not need programming knowledge to run. All of these features make our platform implementable in most routine hematologic laboratories running under time- and cost-effective conditions, independently of the available resources and number of cases per week and/or month to analyze.

Here, we performed the first one-to-one comparison not only with the karyotype or FISH, as in previously published strategies [37,38], but also with SNP arrays, which constitute an extremely high-resolution method for the diagnosis of MDS [1,23]. When clinically relevant CNAs were considered [13,16], our NGS platform demonstrated an approximately 90% concordance with conventional cytogenetics. It is of note that this sensitivity remains unchanged in the comparison with SNP arrays. The few discrepancies observed with conventional cytogenetics and SNP arrays could be mainly classified into two categories. In all (i.e., two del(20q), two del(12p), and one del(17p)) but two cases, unlike DECoN and Ginkgo, B allele frequency plots detected some putative lesions within the involved genomic regions (Figure S4), suggesting that further analysis should be performed for validation. Therefore, all (100%) of these alterations were confirmed by FISH, bringing the sensitivity to ~97% (59 out of 61 events). In the remaining two cases, the discrepancy might be related to the subclonal state of the lesions (i.e., trisomy 8 and del(1)(p10)), since they were uniquely identified by karyotype in only 10% of the metaphases and by FISH in less than 7.5% of the nuclei. However, it should be noted that our NGS approach—as SNP arrays—was able to confirm another subclonal alteration: a trisomy 21 found in an MDS/MPN patient in 2 out of 20 metaphases and 6% of nuclei. All of these findings indicate that our platform has at least the same sensitivity as that of SNP arrays, and is able to detect almost all of the clinically relevant CNAs involving more than 20% of cells. It is of note that this ability was preserved when 50 and 75% random-read subsampling was performed on a selection of 10 specimens (Table S15), obtaining 100% concordance with previously identified CNAs and cnLOH (*n* = 17, Table S16). 

The concordance between our NGS strategy and SNP arrays fell to 67% when CNAs with unknown significance were studied. However, as for clinically relevant CNAs, the BAF plots were able to suggest five additional lesions within the involved regions, bringing the concordance with SNP arrays to 82% (27 out of 33 events). In no cases would the detection of CNAs with unknown significance lead to a change in the cytogenetic risk classification, as these events are mainly seen in an already complex karyotypic background.

Strikingly, compared to conventional cytogenetics, our approach was also able to capture rare events that were described to influence patient prognosis, such as chromotripsis and uniparental disomy. Chromotripsis has been previously reported in myeloid neoplasms and has been associated with adverse outcomes, frequently as a result of concurrent *TP53* mutations [42,43]. In our cohort of MDS and CMML patients, this catastrophic genomic event involved short arms of chromosomes 6, 12, 16, and 19, as well as 18q, whereas chromotripsis of 21q was disregarded as trisomy 21 in an MDS/MPN patient. Instead, all of the cnLOH events were correctly identified (100% concordance) by our strategy. The copy-neutral LOH of 2p, 4q, 7q, and 17p uncovered homozygous mutations of *DNMT3A* P715L, *TET2* Q744X, *EZH2* Y607H, and *TP53* H47Y, respectively. In addition, this marker of clonality was observed in eight patients with normal karyotypes, resulting in an upstaging to the high-cytogenetic-risk group in the case of those harboring cnLOH of 7q and 17p [44].

As shown in this study, an additional advantage of our NGS platform is the assessment of the allelic state of a gene and of the co-occurrence with other genomic lesions. It is of note that, in 11 patients with reported del(5q), our design detected *TP53* mutations in eight of them, and in four cases, these mutations co-occurred with 17p deletion or cnLOH. This information should be included in the clinical report [45], since bi-allelic *TP53* inactivation predicts the risk of death and leukemic transformation independently of the IPSS-R in MDS, and has also been associated with resistance to lenalidomide in del(5q) patients [46,47].

In addition, the identified CNAs and somatic mutations were used as a proof of concept to classify patients according to the established prognostic systems [13,16] and their molecular versions [8,17] by replacing conventional cytogenetics with NGS findings. As expected, the higher number of clinically relevant CNAs detected by NGS led to a change in the prognostic classification, mainly affecting the good cytogenetic and lower-risk groups, which commonly shifted to higher-risk categories. This concerned roughly a quarter and a half of MDS patients when IPSS-R and IPSS-Rm scores were performed with NGS data, respectively, and ~40% of CMML patients. Although further validation should be performed, these results suggest the effectiveness of our platform in prognostic profiling in combination with conventional cytogenetics, as previously demonstrated for FISH [48] and SNP arrays [23].

NGS strategies can represent the future of the diagnosis and prognosis of MDS and related neoplasms. However, the genetic complexity of these disorders has often hampered the translation of NGS into clinical practices. On the one hand, targeted sequencing assays are informative only for selected regions and provide limited information on CNAs and cnLOH. On the other hand, whole-genome sequencing (WGS) can provide information on both, but at the expense of a higher cost and the requirement of complex data storage and analysis methods. Through our NGS-based platform, we tried to overcome the limits of both targeted and whole-genome sequencing, with the advantage of identifying all of the clinically important alterations at a sustainable time and cost (~50–75% less than WGS), thus facilitating data management in terms of storage and complexity of bioinformatic analysis. Based on these features, NGS can likely be integrated into the current diagnostic and prognostic algorithms in a short/medium term. 

## 5. Conclusions

We showed a novel NGS-based platform that is able to identify most of the clinically relevant genetic alterations found in MDS and related neoplasms. This assay covers somatic mutations within significant driver genes and cnLOH events and CNAs distributed genome-wide. It reaches a sensitivity that is at least equivalent to that of SNP arrays and higher than that of conventional cytogenetics. Hence, this approach represents a significant improvement on current strategies in defining the diagnosis and risk stratification of these malignancies.

## Figures and Tables

**Table 1:** Clinical variables of patients included in the study.

Features	N. of Cases/Median	Range
Sex, male/female	36/24	
Age, median	71.5	32–89
BM blast %, median	4	0–18
**Peripheral blood counts**	**Median**	**Range**
Hemoglobin (g/dL)	10	4–13.6
Platelets (×10^9^/L)	120	7–652
Absolute neutrophils count (×10^9^/L)	2.42	0.3–63.4
**2016 WHO classification [1,2]**	**N. of cases**	**Frequency (%)**
MDS with single lineage dysplasia (MDS-SLD)	3	5
MDS with multilineage dysplasia (MDS-MLD)	7	11.7
MDS-RS with single lineage dysplasia (MDS-RS-SLD)	12	20
MDS with isolated del(5q)	4	6.7
MDS with excess blasts (EB)-1	8	13.3
MDS-EB-2	12	20
MDS, unclassifiable (MDS-U)	2	3.3
Myelodysplastic/myeloproliferative neoplasms (MDS/MPN)	2	3.3
Chronic myelomonocytic leukemia (CMML)	10	16.7
**Conventional cytogenetics**	**N. of cases**	**Frequency (%)**
Normal karyotype	37	61.7
Altered Karyotype	22	36.7
NA	1	1.6
**IPSS-R classification [13]**	**N. of cases**	**Risk score**
Very Low	12	1
Low	12	2–3
Intermediate	11	3.5–4
High	5	5–6
Very High	8	6.5–10
NA	2	
**CPSS classification [16]**	**Number of cases**	**Risk score**
Low	1	0
Intermediate-1	2	1
Intermediate-2	7	2–3
High	0	

Abbreviations: N., number; BM, bone marrow; NA, not available; IPSS-R, Revised International Prognostic Scoring System; CPSS, CMML-specific prognostic scoring system.

**Table 2:** CNA and cnLOH events identified by using three different approaches.

CNAs and cnLOH events	Conventional Cytogenetics	SNP-Arrays	DECoN	Ginkgo	NGS-Combined	Uniquely by SNP Arrays	Uniquely by NGS
**Normal karyotype**	**37**	**28**	**30**	**31**	**29**		
**Clinically relevant CNAs**							
-Y	1	1	1	1	1		
del(5q)	8	11	11	9	11	#151908, 63.8 Mb (only by DECoN); #144969, 5.6 Mb (only by DECoN)	
del(12p)	1	5	3	3	3	#163259, 8 Mb; #162250, 7.9 Mb *	
del(20q)	1	3	1	1	1	#140104, 16.7 Mb; #171224, 27.4 Mb	
monosomy 7	8	4	4	4	4		
del(7q)	0	5	5	4	5	#144206, 9.9 Mb(only by DECoN)	
trisomy 8	2	1	1	1	1		
trisomy 21	2	1	1	2	2	#173419, 32.8 Mb (only by Ginkgo)	#144969, trisomy 21
del(17p), i(17)(q10)	3	4	3	2	3	#144969, 21 Mb; #144304, 4.9 Mb (only by DECoN)	
	**26**	**35**	**30**	**27**	**31**		
**Recurrent CNAs with unknown significance**							
del(1p)	1	0	0	0	0		
dup(1p)	0	1	1	0	1		
dup(2p)	1	1	1	0	1		
monosomy 3	1	0	0	0	0		
del(3p)	0	1	1	1	1		
del(5p)	0	1	0	0	0	#144304, 3.3 Mb	
dup(5p)	0	1	0	0	0	#160705, 5.6 Mb	
dup(5q)	1	0	0	0	0		
del(6p)	0	1 ^$^	1 ^$^	1 ^$^	1 ^$^		
dup(6p)	0	1 ^$^	1 ^$^	1 ^$^	1 ^$^		
del(7p)	0	2	1	1	1	#160990, 7.8 Mb (mosaic loss)	
dup(7q)	1	1	0	0	0	#154667, 9.4 Mb	
dup(9p)	0	0	0	1	1		#161780, 5.4 Mb
monosomy 10	1	0	0	0	0		
dup(10p)	0	1	0	1	1	#144969, 36.2 Mb (only by Ginkgo)	
del(10q)	0	1	0	1	1	#144969, 46 Mb (only by Ginkgo)	
dup(11q)	0	1	1	1	1		
dup(12p)	1	0	1	1	1		#162250, 12.9 Mb *
del(12q)	0	2 ^$^	1 ^$^	1 ^$^	1 ^$^	#151908, 9.8 Mb	
dup(13q)	2	0	0	0	0		
dup(15p)	1	1	0	0	0	#151908, 7.6 Mb	
del(16p)	2	3	3	3	3		
dup(16p)	0	1 ^$^	1 ^$^	1 ^$^	1 ^$^		
dup(16q)	1	0	0	0	0		
del(17q)	1	1	0	0	0	#160990, 0.6 Mb	
monosomy 18	2	0	0	0	0		
del(18q)	0	2 ^$^	1 ^$^	2 ^$^	2 ^$^	#144969, 10.3 Mb (only by Ginkgo)	
monosomy 19	1	0	0	0	0		
del(19p)	0	2	0	0	0	#144206, 6.5 Mb; #144969, 7.5 Mb	
dup(19p)	0	2	1	0	1	#151908, 10 Mb; #144969, 6 Mb (only by DECoN)	
dup(19q)	0	1 ^$^	1 ^$^	0	1 ^$^	#151908, 26.7 Mb (only by DECoN)	
monosomy 20	1	0	0	0	0		
dup(20p)	0	1	0	0	0	#151908, 21.8 Mb	
del(21q)	0	1	1	0	1	#162103, 3.2 Mb (only by DECoN)	
dup(21q)	0	2 ^$^	0	0	0	#151908, 8.5 Mb	#144969, trisomy 21
+Y	0	1	1	1	1		
	**17**	**23**	**12**	**11**	**15**		
**Copy-neutral LOH**							
1p cnLOH	NA	1	1	1	1		
1q cnLOH	NA	1	1	1	1		
2p cnLOH	NA	1	1	1	1		
3q cnLOH	NA	1	1	1	1		
4q cnLOH	NA	2	2	2	2		
7q cnLOH	NA	4	4	4	4		
13q cnLOH	NA	1	1	1	1		
17p cnLOH	NA	1	1	1	1		
20p cnLOH	NA	1	1	1	1		
20q cnLOH	NA	1	1	1	1		
	**NA**	**14**	**14**	**14**	**14**		
**TOTAL REPORTED LESIONS**	**43**	**72**	**56**	**52**	**60**		

^$^ chromotripsis; * CNAs were differently interpreted. Abbreviations: CNAs, copy number alterations; SNP, single-nucleotide polymorphism; NGS, next-generation sequencing; cnLOH, copy-neutral loss of heterozygosity; Del, deletion; Dup, duplication.

## Data Availability

Raw sequencing and SNP array data will be available by request from the corresponding authors.

## Supplementary Materials

The following are available online at https://www.mdpi.com/article/10.3390/cancers13081947/s1, Table S1: Custom-design targeted regions on a Human Feb. 2009 (GRCh37/hg19) Assembly, Table S2: Depth of coverage per patient for each targeted region in the design, Table S3: List of somatic variants identified in the cohort and classified as pathogenic or likely pathogenic, Table S4: List of SNPs used to compute B allele frequency, Table S5: One-to-one comparison of CNAs identified in NA12144 cell lines by our NGS platform with those reported in a previous publication [49], Table S6: List of significant pairwise gene associations, Table S7: List of CNAs and cnLOH events detected in the study, Table S8: Somatic cytogenetic lesions identified by using SNP arrays, Table S9: List of clinically significant CNAs identified by DECoN, Table S10: List of CNAs identified by Ginkgo, Table S11: List of cnLOH identified by NGS and confirmed by SNP arrays, Table S12: Correlation between CNAs and molecular alterations detected at the same genomic region, Table S13: Tumor burden and breakpoint definition of del(5q) and chromosome 7 alterations, Table S14: Patient classification according to the standard and molecular versions of IPSS-R and CPSS scoring systems, Table S15: Average coverage at 100%, 50%, and 25% of available reads, Table S16: Comparative of CNAs identified by Ginkgo at 100%, 50%, and 25% of available reads, Figure S1: Two cases of discrepancy in CNA detection between SNP arrays and NGS. Snapshots of the “*karyogram*” from SNP arrays and “*profile view*” from Ginkgo are shown for chromosomes 12 and 21. At the top, a telomeric duplication (12p13.33-p13.31, 7.5 Mb) and a flanking deletion (12p13.31-p12.3, 7.9 Mb), including the *ETV6* gene, were identified exclusively by the NGS and SNP arrays, respectively. At the bottom, NGS identified a trisomy 21, whereas the SNP arrays detected a dup(21q21.2-q22.3) interspaced by a 1-Mb 21q21.3 deletion, Figure S2: Chromotripsis identified in a high-risk MDS patient. Chromotripsis of chromosomes 12p, 12q, 16p, and 18q can be observed both within the Circos graphic (i.e., NGS, in the left panel) and “*karyogram*” (i.e., SNP arrays, in the right panel). Gains appear in blue, deletions in red, and cnLOH in violet, Figure S3: Copy-neutral loss of heterozygosity events identified by SNP arrays and NGS. For each cnLOH event, a chart is shown, which includes the patient’s ID and the genomic region harboring the alteration (at the top of the chart). In addition, a “*karyogram*” of the involved chromosome is placed above the BAF plots as a result of the SNP arrays and NGS analysis, respectively. Figure S4: Discrepancies in CNA detection between SNP arrays and NGS solved with the BAF plots. A snapshot of the “*karyogram*” obtained from SNP arrays and the BAF plots from the NGS assay are shown for two deletions in chromosomes 12 and 20, and another one in chromosome 17. Unlike DECoN and Ginkgo, the BAF plots detect some putative lesions within the same regions where SNP arrays have identified loss of chromosome material.
