# Supplementary material for: A Single-Run Next-Generation Sequencing (NGS) Assay for the Simultaneous Detection of Both Gene Mutations and Large Chromosomal Abnormalities in Patients with Myelodysplastic Syndromes (MDS) and Related Myeloid Neoplasms
Source: Cancers (Basel). 2021 Apr 18;13(8):1947. doi: 10.3390/cancers13081947 (PMC8072643; doi:10.3390/cancers13081947)
Supplement: Supplementary file 1 [file cancers-13-01947-s001.zip › cancers-1174507- supplementary materials/Figures S1-4.pdf]

Supplementary Materials: A Single-Run Next-Generation Sequencing (NGS) Assay for the Simultaneous Detection of Both Gene Mutations and Large Chromosomal Abnormalities in Patients with Myelodysplastic Syndromes (MDS) and Related Myeloid Neoplasms

Alessandro Liquori, Iván Lesende, Laura Palomo, Gayane Avetisyan, Mariam Ibáñez, Elisa González-Romero, Mireia Boluda-Navarro, Mireya Morote-Faubel, Cristian Garcia-Ruiz, Cristina Martinez-Valiente, Marta Santiago-Balsera, Inés Gomez-Seguí, Alejandra Sanjuan-Pla, Miguel A. Sanz, Guillermo Sanz, Francesc Solé, Esperanza Such and José Cervera

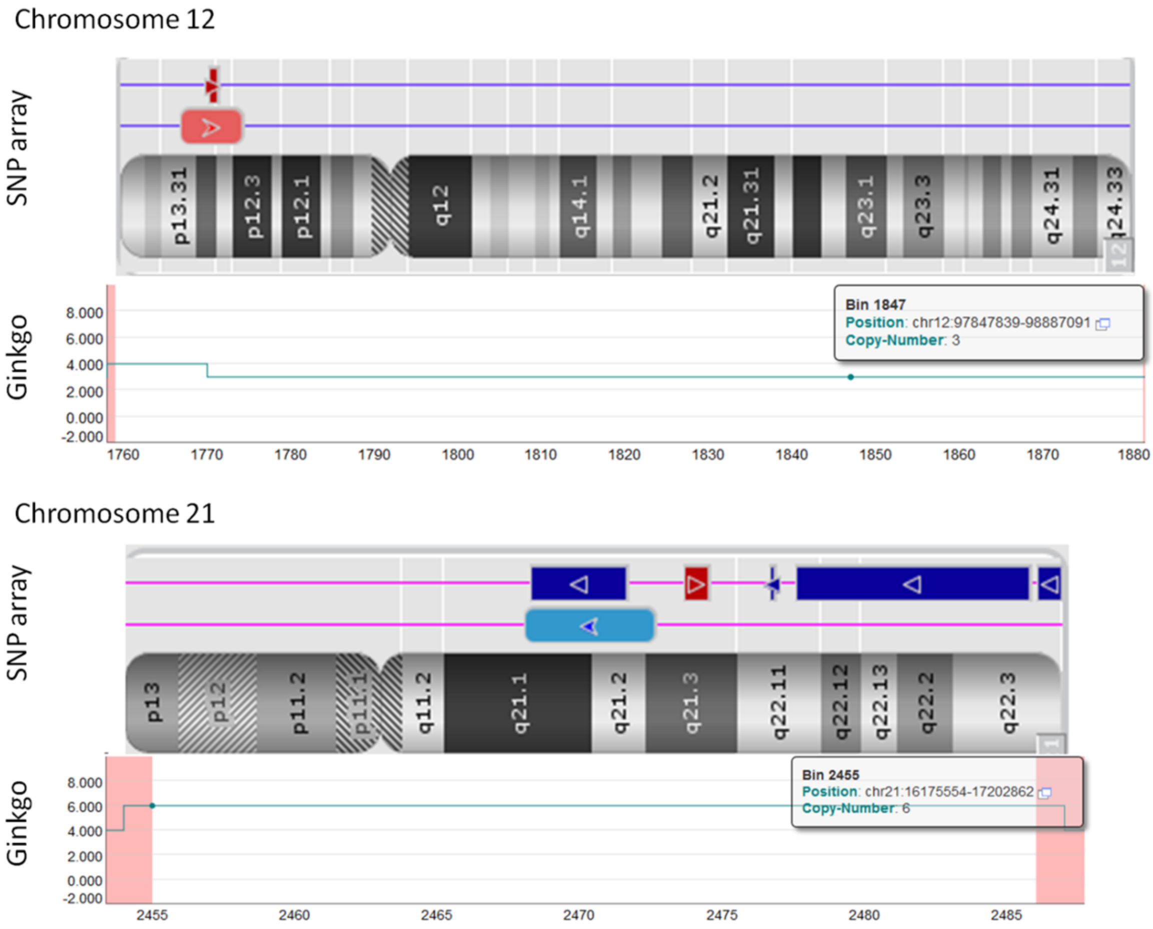

Figure S1. Two cases of discrepancy in CNAs detection between SNP array and NGS. A snapshot of the "karyogram" from SNP arrays and "profile view" from Ginkgo are shown for chromosome 12 and 21. At the top, a telomeric duplication (12p13.33-p13.31, 7.5 Mb) and a flanking deletion (12p13.31-p12.3, 7.9 Mb), including the ETV6 gene, were identified exclusively by NGS and SNP arrays, respectively. At the bottom, NGS identified a trisomy 21 whereas SNP arrays detected a dup(21q21.2-q22.3) interspaced by a 1-Mb 21q21.3 deletion.

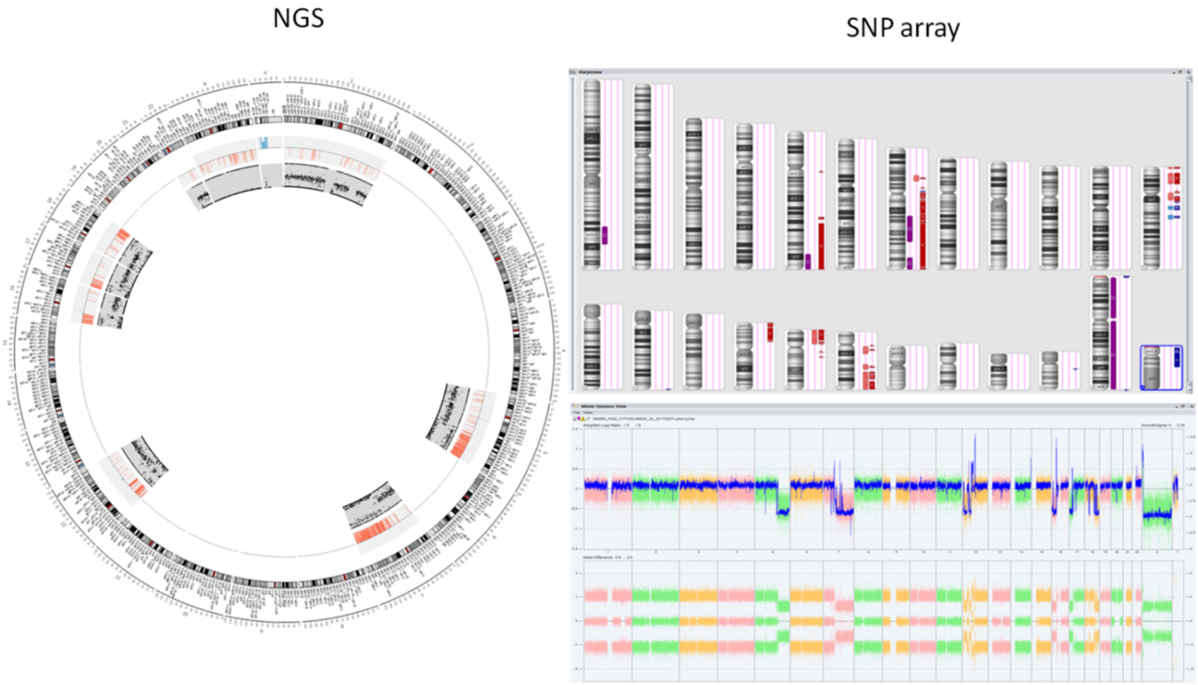

Figure S2. Chromotripsis identified in a high-risk MDS patient. Chromotripsis of chromosomes 12p, 12q, 16p and 18q can be observed both within circos graphic (i.e., NGS, at the left panel) and “karyogram” (i.e., SNP arrays, at the right panel). Gains appear in blue, deletions in red and cnLOH in violet.

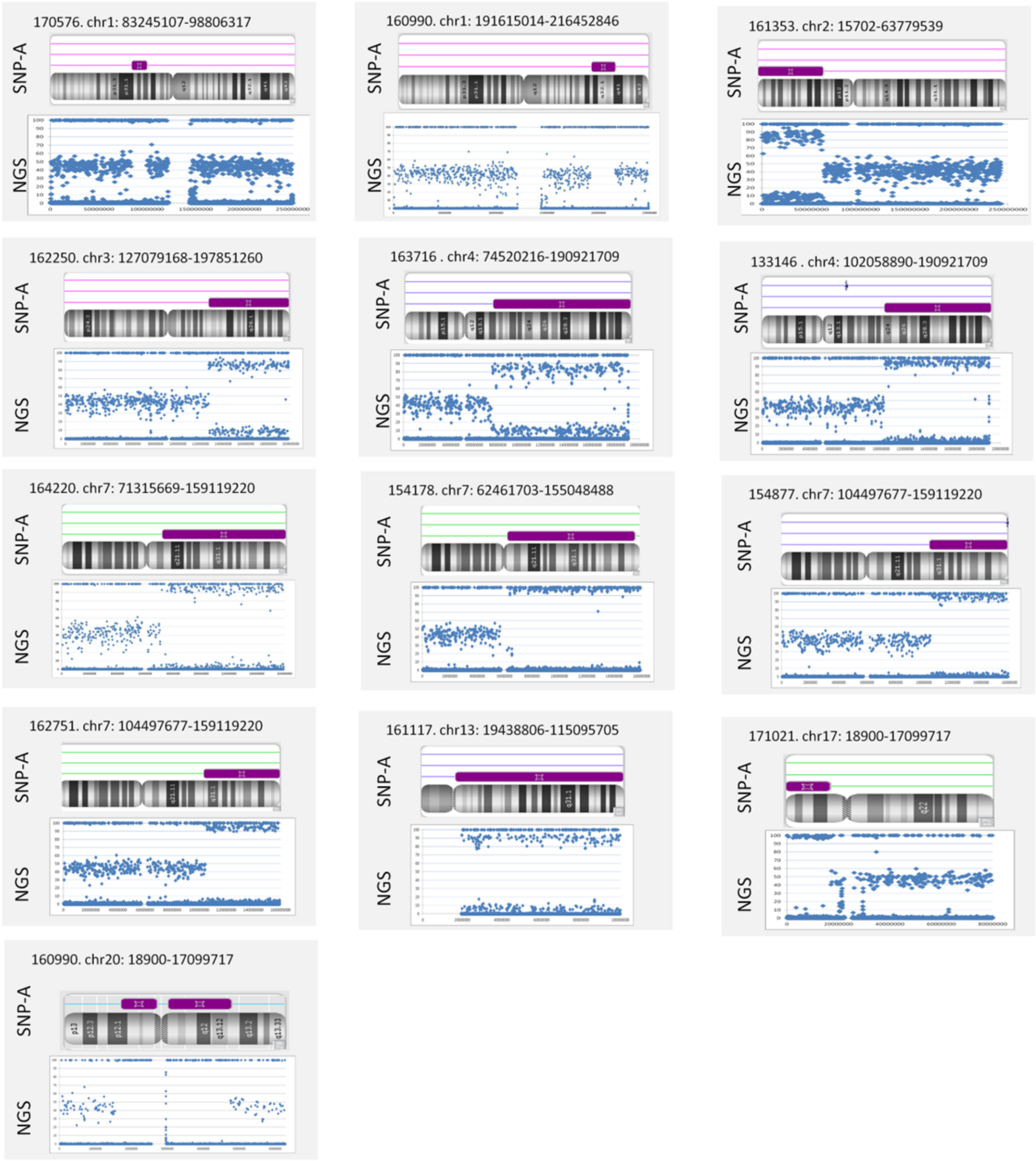

Figure S3. Copy-neutral loss of heterozygosity events identified by SNP arrays and NGS. For each cnLOH event a chart is shown including the patient's ID and the genomic region harboring the alteration (at the top of the chart). In addition, a "karyogram" of the involved chromosome is placed above BAF plots as a result of SNP arrays and NGS analysis, respectively.

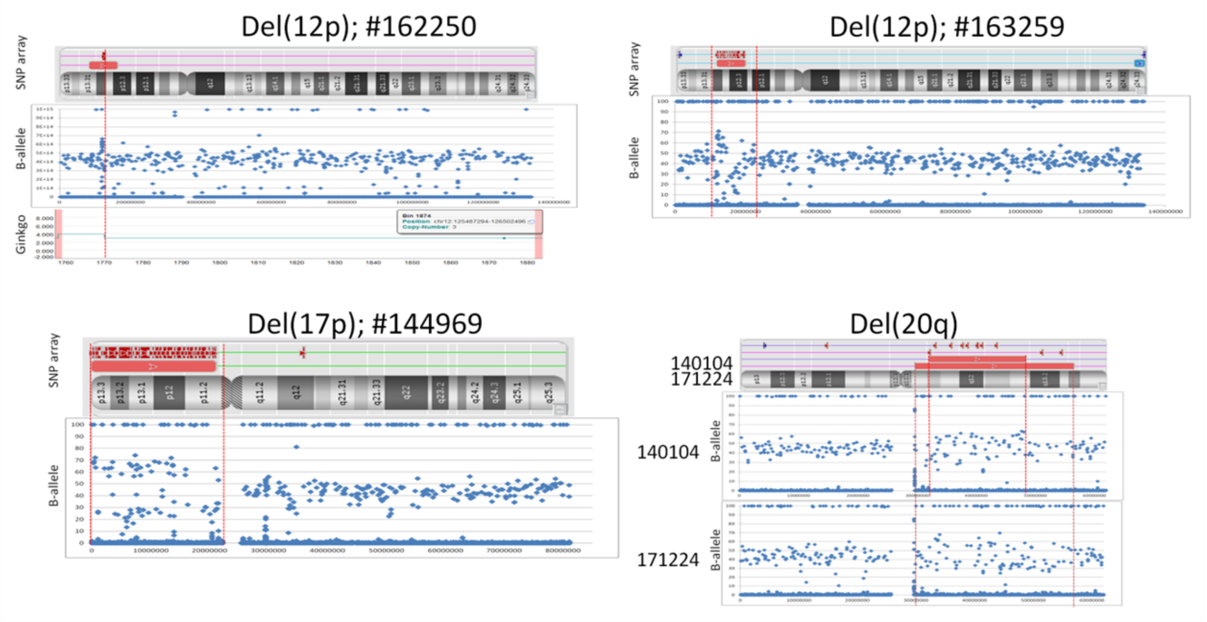

Figure S4. Discrepancies in CNAs detection between SNP arrays and NGS solved by BAF plots. A snapshot of the "karyogram" obtained from SNP arrays and BAF plots from NGS assay is shown for two deletions in chromosomes 12 and 20, and another one in chromosome 17. Unlike DECoN and Ginkgo, BAF plots detect some putative lesions within the same regions where SNP arrays have identified loss of chromosome material.

Table S1–16. please view at excel file.
